# Higher preoperative pain catastrophizing increases the risk of low patient reported satisfaction after carpal tunnel release: a prospective study

**DOI:** 10.1186/s12891-020-3058-2

**Published:** 2020-01-18

**Authors:** Sebastian Breddam Mosegaard, Maiken Stilling, Torben Bæk Hansen

**Affiliations:** 1 Department of Orthopaedics, University Clinic for Hand, Hip and Knee Surgery, Lægårdvej 12, 7500 Holstebro, Denmark; 2 Department of Clinical Medicine, Aarhus University, 8000 Aarhus, Denmark

**Keywords:** Carpal tunnel syndrome, Pain catastrophizing scale, Patient satisfaction, Risk factors

## Abstract

**Background:**

Carpal tunnel syndrome is a common upper-limb nerve compression disease. Carpal tunnel syndrome can lead to several symptoms such as tingling or numbness, pain in the hand or wrist, and reduced grip strength. Based on demographic characteristics, patient reported outcome measures, and with special attention to pain catastrophizing, the purpose of this study was to identify risk factors for low patient-reported satisfaction following surgical treatment of idiopathic carpal tunnel syndrome.

**Methods:**

A total of 417 hands from 417 patients (64. 5% females) with a mean age of 58. 0 years were included in this 1-year prospective follow-up study. We collected preoperative data on disability using the Disability of the Arm, Shoulder and Hand questionnaire (DASH), quality of life using the EuroQol-5D (EQ-5D), pain catastrophizing using the Pain Catastrophizing Scale (PCS) and distal motor latency. Data on DASH score, EQ-5D, and patient satisfaction was collected 12 months postoperatively. Wilcoxon matched-pairs signed-rank test was used to test for difference in preoperative and postoperative DASH and EQ-5D score. Risk factors for low postoperative patient reported satisfaction was examined using stepwise multiple logistic regression analysis.

**Results:**

We found a general improvement in patients’ DASH scores (12.29 [95% CI: 10.65–13.90], *p* < 0.001) and EQ-5D (0.14 [95% CI: 0.13–0.16], *p* < 0.001) from preoperative to 12 months postoperative. In the fully adjusted multiple regression analysis we found a statistically significant effect of preoperative PCS on patient reported satisfaction with OR = 1.05 (*p* = 0.022), for a one unit increase in preoperative PCS. There was no statistically significant predictive effect of preoperative EQ-5D (*p* = 0.869), DASH (*p* = 0.076), distal motor latency (*p* = 0.067), age (*p* = 0.505) or gender (*p* = 0.222).

**Conclusions:**

Patients improved in both DASH and EQ-5D from preoperative to 12 months postoperative. Higher preoperative PCS seems to have a negative effect on postoperative patient reported satisfaction after carpal tunnel release.

## Background

Idiopathic carpal tunnel syndrome (CTS) is a common upper limb nerve compression disease [1]. CTS can lead to several symptoms such as tingling or numbness, pain, and reduced grip strength [2]. It appears mainly in middle-aged women [1, 3, 4], with an approximated gender ratio of 3:1 [5]. The European prevalence is estimated to be 1–7% [5, 6], with an incidence of 1.8 per 1000 years [5], leading to roughly 300,000 operations per year in Germany [7]. Emphasizing the incidence of CTS, it is estimated that close to 1 million people annually need medical treatment of CTS in America [3]. Surgical decompression with either endoscopic carpal tunnel release (ECTR) or open carpal tunnel release (OCTR) is used to improve function and relieve symptoms [8] when conservative treatment (steroid injections and orthoses) of the hand is inadequate [9]. Although the outcome following carpal tunnel release (CTR) is mainly positive, symptoms remain or reoccur in 3–20% of cases [10, 11]. Several factors have been suggested to be predictive of negative surgical outcomes; smoking, bilateral CTS, low preoperative symptom severity, diabetes, older age, poor physical health, and poor mental health [12–14].

The use of patient reported outcome measures (PROMs) to evaluate the surgical outcome has increased. Furthermore, the overall patient satisfaction has been shown to predict the sick leave duration following CTR [15]. In a systematic review from 2017, 3 of 5 studies showed a significant correlation between patient satisfaction and psychological measures of depression and mental health in CTS patients [16]. Studies further show that self-reported depression is correlated to poorer self-evaluated hand function in patients suffering from trapeziometacarpal arthritis [17]. In CTS patients, preoperative hospital anxiety is associated with worse preoperative symptom severity [18]. Additionally, a worse score on the 5-item Mental Health interview has been associated to lower postoperative patient satisfaction [13]. These studies indicate the effect of psychological factors on different outcome measures including satisfaction. However, little attention has been drawn to the effect of pain catastrophizing (measured using the Pain Catastrophizing Scale (PCS)) on patient satisfaction following CTR. A study from 2010 on 120 patients with different hand diseases (carpal tunnel syndrome, trigger finger, and benign tumors) did not find a correlation between preoperative PCS and postoperative DASH scores [19]. Conversely, a newer study from 2014 on 256 patients with atraumatic hand disorders found an association between PCS and the Michigan Hand Outcome Questionnaire (MHOQ). The study showed worse scores on the MHOQ for patients with high PCS (PCS > 30) compared to patients with low PCS (PCS ≤ 30) at baseline, and at 1- and 2-month follow-ups [20].

To our knowledge, only one study has briefly examined the effect of PCS on patient satisfaction in CTS patients [21]. This retrospective study on 82 patients did not find an association between PCS and patient satisfaction in a univariate analysis and did not examine it further. Given the results from other studies indicating an effect of mental health and PCS on the outcome after treatment of hand disorders, this study aimed to further investigate the effect of PCS.

Based on demographic characteristics, PROMs, and with special attention to PCS, the purpose of this study was to identify risk factors for low patient-reported satisfaction following surgical treatment of idiopathic CTS with CTR. The main hypothesis of this study was that higher preoperative PCS scores increase the risk of low postoperative patient reported satisfaction.

## Methods

Patients with nerve conduction verified Carpal Tunnel Syndrome (CTS) were recruited between February 11th 2011 and January 5th 2015 at the Department of Orthopaedics, Regional Hospital Holstebro. This prospective cohort consists of 732 hands from 714 patients treated surgically for CTS with either open carpal tunnel release (OCTR - 38%) or endoscopic carpal tunnel release (ECTR - 62%). Patients were asked to fill out a set of questionnaires preoperatively and 12 months postoperatively. The preoperative questionnaires included; a health-related quality of life assessment using EQ-5D, hand function using a translated and validated version of the Disabilities of the Arm, Shoulder and Hand questionnaire (DASH) [22, 23], and catastrophic thinking of pain using the Pain Catastrophizing Scale (PCS). The DASH questionnaire is a 30-item questionnaire used to measure patient reported disability through 30 statements on a 5-point Likert scale, where a higher score reflects more disability [24]. PCS is used to measure coping skills and negative feelings of pain through 13 statements with 4 possible options from 1 “not at all” to 4 “all the time” with a higher score reflecting higher catastrophic thinking [25]. The score can further be categorized as either high (PCS > 30) or low (PCS ≤ 30) [26]. Distal motor latency was registered following preoperative nerve conduction tests.

The 12-month postoperative questionnaire included the EQ-5D, DASH, and a question on patient satisfaction with 4 options ranging from 1 “I am dissatisfied” to 4 “I am very satisfied”. We then pooled options 1 and 2 as low satisfaction and options 3 and 4 as high satisfaction.

### Patient demographics

All patients with nerve conduction verified CTS were assessed for eligibility (714). The second operated hand was excluded from 18 bilateral patients to avoid statistical dependence, and 92 hands were excluded due to missing preoperative data on DASH, EQ-5D, and PCS. A further 205 hands were excluded due to missing 12-month postoperative data on DASH and EQ-5D, leaving 417 patients (64. 5% women) with a mean age of 58 years (range, 18–92 yrs.) for analysis. The exclusion of patients did not lead to statistically significant changes in preoperative baseline characteristics. Further patient characteristics before and after exclusion can be seen in Table 1.

**Table 1:** Preoperative baseline characteristics with 95% confidence intervals before and after exclusion

	Baseline before exclusion (*N* = 732)		Baseline after exclusion (*N* = 417)	
	Mean	95% CI	Mean	95% CI
Female %	63. 9%	.	64. 5%	.
Age, years	58. 0	56. 9–59. 1	58. 0	56. 5–59. 4
Female age, years	57. 2	55. 8–58. 6	56. 7	54. 9–58. 5
Male age, years	59. 5	57. 5–61. 4	60. 4	58. 0–62. 8
DASH	26. 1	24. 2–27. 9	25. 4	23. 5–27. 4
EQ-5d	0. 74	0. 72–0. 76	0. 74	0. 72–0. 76
PCS	13. 3	12. 3–14. 3	13. 0	11. 9–14. 1
Distal motor latency, m/s	5. 7	5. 6–5. 9	5. 7	5. 5–5. 9

Preoperative data on patients before and after exclusion both preoperative and 12-months postoperative

The study was reviewed by the local research ethics committee, and no further specific approval was demanded because the study is an outcome study, which according to the Danish law,“Act on a Biomedical Research Ethics Committee System and the Processing of Biomedical Research Projects”, Part 3 “Notification and Authorization: Questionnaire-based projects and register research projects shall only be notified to a regional committee if the project also involves human biological material.” The study was registered in The Danish Data Protection Agency: jr. nr.: 2007-58-0010.

### Statistics

Wilcoxon matched-pairs signed-rank test was used to test for difference in preoperative and postoperative DASH and EQ-5D scores due to non-normality. Logistic regression analysis and multiple logistic regression analysis were used to test predictors of low patient reported satisfaction following surgical treatment of CTS in Tables 2 and 3. This was done in four steps. Step 1 was crude logistic regressions of the associations between the variables of interest one by one and the dichotomous outcome high/low satisfaction. Step 2 was to adjust for preoperative baseline characteristics; age, gender, and operation technique. Step 3 was to adjust for age, gender, operation technique, and further adjust for the other predictors of interest; PCS, EQ-5D, DASH, and distal motor latency. The 4th and final step was to examine multicollinearity in the models. We examined multicollinearity in the multivariate logistic regression models using variance inflation factors (VIF), finding no VIF > 2.02. All statistical analyses were made using STATA, Version 15 IC (Stata Corp, College Station, TX, USA).

**Table 2:** Preoperative baseline characteristics for highly satisfied and lowly satisfied patients

	High satisfaction		Low satisfaction	
	Mean	95% CI	Mean	95% CI
Female, %	66. 0%	.	60. 1%	.
Age, years	55. 8	53. 8–57. 7	56. 2	51. 6–60. 9
DASH	22. 7	20. 4–24. 9	38. 0	31. 6–44. 4
Eq-5d	0. 76	0. 74–0. 78	0. 66	0. 59–0. 74
PCS	11. 2	10. 0–12. 4	19. 6	16. 0–23. 3
Distal motor latency, m/s	5. 70	5. 46–5. 93	5. 13	4. 72–5. 56

Preoperative baseline characteristics with mean and 95% confidence intervals for patients reporting high satisfaction and patients reporting low satisfaction 12 months postoperative

**Table 3:** The association between baseline characteristics and patient reported satisfaction

Preoperative	Odds ratio for low patient reported satisfaction following CTR		
	Odds ratio	95% CI	*p*
PCS			
Unadjusted^a^	1. 08	1. 05–1. 11	< 0. 001*
+ Demographics^b^	1. 09	1. 06–1. 12	< 0. 001*
+ Disability^c^	1. 05	1. 01–1. 10	0. 022*
EQ-5D			
Unadjusted^a^	0. 13	0. 03–0. 51	0. 004*
+ Demographics^b^	0. 10	0. 02–0. 46	0. 003*
+ Disability^c^	0. 82	0. 09–7. 82	0. 869
DASH			
Unadjusted^a^	1. 04	1. 02–1. 05	< 0. 001*
+ Demographics^b^	1. 04	1. 02–1. 06	< 0. 001*
+ Disability^c^	1. 02	1. 00–1. 05	0. 056
Distal motor latency			
Unadjusted^a^	0. 83	0. 68–1. 01	0. 063
+ Demographics^b^	0. 78	0. 63–0. 98	0. 030*
+ Disability^c^	0. 75	0. 55–1. 02	0. 067
Living alone			
Unadjusted ^a^	0. 70	0. 35–1. 41	0. 320
+ Demographics ^b^	0. 69	0. 33–1. 44	0. 321
+ Disability ^c^	0. 36	0. 11–1. 81	0. 092

Multiple logistic regression analysis on the association between baseline characteristics and patient reported satisfaction 12 months postoperative

*Denotes statistical significance

^a^Unadjusted crude association on odds ratio for low patient reported satisfaction

^b^Adjusted for age, gender, living alone and operation technique

^c^Adjusted for age, gender, operation technique, living alone and preoperative scores (PCS, EQ-5D, DASH and distal motor latency)

## Results

When analyzing the patients as one group, we found a statistically significant improvement in both DASH and EQ-5D at the 12-month follow-up. The mean improvement in EQ-5D was 0.14 [95% CI: 0.13–0.16] (*p* < 0.001), which was a change from 0.74 [95% CI: 0.72–0.76] preoperatively to 0.89 [95% CI: 0.87–0.91] 12 months postoperatively, which is more than the estimated minimal clinical important difference (MCID) of 0.10 [27]. DASH scores improved by 12.29 [95% CI:10.65–13.90] (*p* < 0.001), which was a change from 24.88 [95% CI:22.87–26.89] preoperatively to 12.60 [95% CI,10.73–14.47] 12 months postoperatively, which is more than the MCID of 12 points for the Danish validated DASH [28].

The patients reporting low satisfaction at 12 months had a higher preoperative PCS score, lower EQ-5D, and higher DASH score. Further, the patients reporting low satisfaction had a tendency toward lower preoperative distal motor latency but with overlapping confidence intervals for the mean. There was no statistical difference in age and gender between patients reporting low satisfaction and patients reporting high satisfaction. Means and confidence intervals can be seen in Table 2.

Table 3 shows the logistic regression models of the association between the possible predictive preoperative variables. After including both demographics (age, gender, operation technique, and living alone) and preoperative disability (PCS, EQ-5D, DASH, and distal motor latency) in the model, we found a statistically significant effect of preoperative PCS on patient reported satisfaction with OR = 1.05 (*p* = 0.022) for a 1-unit increase in preoperative PCS.

We did not find a statistically significant effect of EQ-5D (*p* = 0.869), DASH (*p* = 0.076), distal motor latency (*p* = 0.067), age (*p* = 0.505), or gender (*p* = 0.222). Although the *p*-value related to the preoperative DASH score exceeded the 0.05 significance level, the 95% confidence interval for the odds ratio ranging from [1.00–1.05] indicates that there could be a tendency toward an increased risk of low patient reported satisfaction with an increased preoperative DASH score.

Table 4 shows an analysis of the risk of low satisfaction for patients with preoperative PCS > 30 compared to patients with PCS ≤ 30. Unadjusted and adjusted for demographics, we found an OR of 2.24 and 2.56 respectively for low satisfaction for patients with preoperative PCS > 30 (*p* = 0.005 & *p* = 0.003 respectively). However, when further adjusting for preoperative DASH, EQ-5D, and distal motor latency, the OR dropped to 1.85 [95% CI: 0.78–4.39], and was no longer significant.

**Table 4:** The risk of low patient reported satisfaction for patients with preoperative PCS > 30

Preoperative	Odds ratio for low patient reported satisfaction following CTR		
	Odds ratio	95% CI	*p*
PCS > 30			
Unadjusted ^a^	2. 24	1. 27–3. 96	0. 005*
+ Demographics^b^	2. 56	1. 38–4. 74	0. 003*
+ Disability ^c^	1. 85	0. 78–4. 39	0. 165

Multiple logistic regression analysis on the risk of low patient reported satisfaction 12 months postoperative for patients with preoperative PCS > 30 compared to patients with PCS ≤ 30

*Denotes statistical significance

^a^Unadjusted crude association on odds ratio for low patient reported satisfaction

^b^Adjusted for age, gender, living alone and operation technique

^c^Adjusted for age, gender, operation technique, living alone and preoperative variables (DASH, EQ-5D and Distal motor latency)

**Fig. 1:**
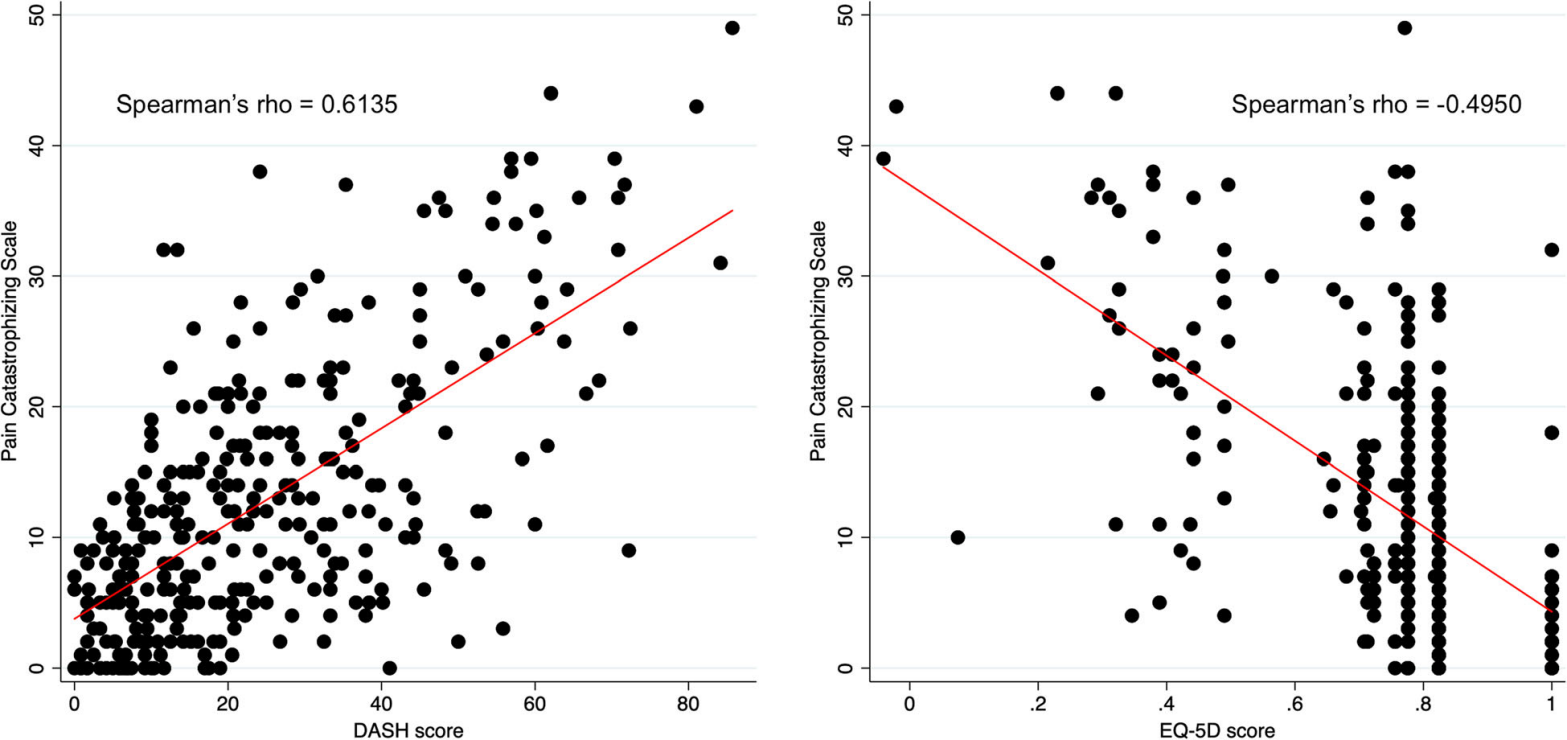
Preoperative PCS against DASH and EQ-5D. Left side: Scatterplot of preoperative PSC and DASH score showing a positive correlation between PCS and DASH score with Spearman’s rho = 0. 6135. The red line illustrates a linear line of best fit. Right side: Scatterplot of preoperative PSC and EQ-5D score showing a negative correlation between PCS and EQ-5D score with Spearman’s rho = − 0. 4950. The red line illustrates a linear line of best fit

Finally, Fig. 1 shows a scatter plot of the preoperative PCS and the preoperative DASH and EQ-5D scores. Correlation analysis using Spearman’s rho shows a correlation of rho = 0.6135 (*p* < 0.001) and rho = − 0.4950 (*p* < 0.001) for PCS and DASH, and PCS and EQ-5D respectively. This indicate that the patients with high preoperative PCS tend to score worse on both preoperative DASH and EQ-5D.

Since DASH and EQ-5D were collected both at baseline and 12 months postoperatively, we analyzed the effect of improvement in DASH and EQ-5D on the patient reported satisfaction after CTR. In the fully adjusted models, we found an OR for low patient reported satisfaction of 0.93 for a 1-unit increased improvement in DASH (*p* < 0.001) and an OR of 0.54 for a 0.1-unit increased improvement in EQ-5D (*p* < 0.001). For both DASH and EQ-5D, it shows that the risk of low patient reported satisfaction is reduced with increased improvement.

## Discussion

We found a significant improvement above the MCIDs in patient disability measured by DASH, and in quality of life measured by EQ-5D, from baseline to the 12-month follow-up after CTR. A total of 84.2% of the patients felt either satisfied or very satisfied 12 months after the operation. Higher preoperative PCS had a statistically significant negative influence on patient reported satisfaction. Furthermore, we found a trend towards a negative predictive effect of low preoperative distal motor latency and a high preoperative DASH score. There was no predictive effect of age, gender, or preoperative EQ-5D, on postoperative patient satisfaction.

In secondary analyses, we found that lower improvements in both postoperative DASH and EQ-5D increased the risk of low patient reported satisfaction, Table 5.

**Table 5:** The association of change in DASH and EQ-5D and low patient reported satisfaction

Change	Odds ratio for low patient reported satisfaction following CTR		
	Odds ratio	95% CI	*p*
DASH ^d^			
Unadjusted	0. 93	0. 90–0. 96	< 0. 001*
+ Demographics ^a^	0. 93	0. 91–0. 96	< 0. 001*
+ Disability ^b^	0. 92	0. 89–0. 95	< 0. 001*
EQ 5D ^e^			
Unadjusted	0. 54	0. 43–0. 67	< 0. 001*
+ Demographics ^a^	0. 54	0. 43–0. 68	< 0. 001*
+ Disability ^c^	0. 53	0. 40–0. 69	< 0. 001*

Multiple logistic regression analysis on the association of change in DASH and EQ-5D and low patient reported satisfaction 12 months postoperative

*Denotes statistical significance

^a^Unadjusted crude association on odds ratio for low patient reported satisfaction

^b^Adjusted for age, gender, living alone and operation technique

^c^Adjusted for age, gender, operation technique, living alone and preoperative variables (PCS, EQ-5D and distal motor latency)

^d^The effect of a 1- unit increase in DASH improvement

^e^The effect of a 0. 1- unit increase in Eq-5d improvement

### Age and gender

Although we did not find age and gender to be a predictor of patient satisfaction, previous studies have shown diverse findings. In 1998, Atroshi et al. found higher age to be a risk factor for low patient reported satisfaction 6 months after OCTR in a study on 128 Swedish patients (mean age 51 years, range 21–94) [29]. On the contrary, a Taiwanese study including 58 patients (mean age 50.6 years, SD = 10.54) did not find a predictive effect of age on postoperative patient satisfaction [30]. The effect of age has also been examined with other outcomes such as return to work, disability, and symptom relief, with mixed findings showing no effect of age on return to work [31], QuickDASH improvement [12], or disability [32]. But higher age has been found to have a negative effect on symptom relief 6 months after CTR [33].

As with age, we did not find a similar effect of gender on patient satisfaction as Atroshi et al. [29]. However, a Danish prospective cohort study on 101 patients did find males to be less satisfied than females 2 months after ECTR [34]. Additionally, gender had no effect in studies of return to work [31], QuickDASH improvement [12], disability [32], or symptom relief [33]. With the mixed results from this study and previous studies in mind, the effects of both age and gender still seem unclear.

### Distal motor latency

We found that lower preoperative distal motor latency might increase the risk of low patient reported satisfaction 12 months after CTR. The same has been shown in a study measuring patient satisfaction 6 months after CTR [29], indicating that preoperative distal motor latency could be a valuable tool in predicting postoperative patient satisfaction. This may also reflect that patients with low distal motor latency have less to gain after an operation compared to patients with more severe nerve compression. Conversely, a Danish study on 75 patients found that higher distal motor latency, indicating more severe median nerve compression, led to longer sick leave from work following CTR [35].

### PCS

In this study, preoperative PCS was found to have a predictive effect on the 12-month postoperative patient satisfaction, with a higher (worse) PCS increasing the risk of low postoperative patient satisfaction. Dissimilar to our results, a retrospective study from 2008 with comparable age and gender distribution on 82 (53 women / 29 men) American patients (mean age 61 years, range 34–92), did not find a correlation between PCS and patient satisfaction after a minimum of 2 years [21]. Additionally, another American study on 120 patients (69 women / 51 men) with a mean age of 61 years (range 18–86), showed no correlation between postoperative PCS and DASH, but a correlation between PCS and pain at the time of suture removal (10–14 days after surgery) in a cohort of patients treated for CTS (*n* = 39), trigger finger (*n* = 65) and benign tumors (*n* = 16) [19]. This difference in results may reflect the different study designs and number of patients.

The effects of other psychological measures have been examined in previous studies with various results. “The Hospital Anxiety and Depression Score” (HADS), is a reliable instrument used to detect and evaluate severity of depression and anxiety [36]. An English study from 2005 showed no difference in patient satisfaction and Boston Carpal Tunnel Questionnaire (BCTQ) between patients with high and low HADS 6 months after CTR surgery [18]. Mental health status measured by subscales from the SF-36 has shown that worse mental health status predicts lower postoperative patient satisfaction 18 months after CTR [13]. Similarly, a 13-study systematic review found that a worse mental health status leads to longer sick leave after CTR [37]. Additionally, a weak correlation between depression and patient satisfaction was shown in an 8-study systematic review [16], where 3 in 5 studies on patients treated with CTR established a significant negative association between patient satisfaction and psychological factors measured using the Centers of the Epidemiological Study of Depression Instrument (CES-D), 5-item Mental Health Interview, and HADS.

The results from the present study indicate a predictive negative effect of higher preoperative PCS on patient reported satisfaction 12 months after CTR. If possible, clinicians should examine both the patient’s physical- and mental health status and discuss these parameters with the patient before performing CTR. PCS might be a useful tool for doing so even though this study did not find a statistically significant increased risk when dividing patients in PCS groups using a score ≥ 30 as the cutoff value [26]. We believe that these results call for further studies on the predictive effects of PCS.

### Considerations

We used the DASH score as a measure of patient disability. Since DASH targets both the arm, shoulder and hand, other injuries not related to CTS might affect the validity of DASH as an instrument to measure disability related to CTS. The use of a CTS related disability questionnaire e.g. the Boston Carpal Tunnel Questionnaire, might have increased the accuracy of the measurements.

Another consideration is the exclusion of 315 (43%) patients due to missing data. 43% is a large number of excluded patients, which potentially could lead to bias. We did however not see a change in baseline characteristics after exclusion of patients without a full dataset.

Several other factors, which were not investigated in this study, such as lower income [31], active smoking status [12, 38], and higher alcohol consumption [13, 38], have been shown to negatively affect the patients’ outcome after CTR. Therefore, it would be of great interest to include these in statistical models on the predictive effect of PCS on patient satisfaction.

We used a 4-item Likert scale to examine patient satisfaction using one statement. An English study with 810 patients examined “The Friends and Family Test” (FFT), which is a variation of the “Net Promoter Score” (NPS) used to measure overall patient satisfaction. They found the FFT to be correlated to patient satisfaction, hospital experience, and functional outcome [39]. It would be interesting to examine the possible predictive effect of PCS on FFT. Given FFT’s correlation to both satisfaction and function, a possible association between PCS and FFT would enable practitioners and surgeons to counsel the patient’s potential outcome after CTR, not only based on satisfaction, but also as a surrogate marker of functional outcome.

## Conclusion

CTR is an effective treatment for carpal tunnel syndrome with high patient satisfaction and improvement after 12 months in both DASH score and EQ-5D. Higher preoperative PCS seems to have a negative effect on postoperative patient reported satisfaction after CTR. Further studies on patient satisfaction should include additional information on patient smoking habits, alcohol consumption, BMI, diabetes, and income, to strengthen the explanatory power.

## Data Availability

The datasets used and analyzed during the current study are available from the corresponding author on reasonable request.

## Abbreviations

BCTQ: Boston Carpal Tunnel Questionnaire
CES-D: Centers of the Epidemiological Study of Depression Instrument
CTR: Carpal tunnel release
CTS: Carpal tunnel syndrome
DASH: Disabilities of the Arm Shoulder and Hand questionnaire
ECTR: Endoscopic carpal tunnel release
FFT: The Friends and Family Test
HADS: Hospital anxiety and depression scale
MCID: Minimal clinical important difference
MHOQ: Michigan Hand Outcome Questionnaire
NPS: Net Promotor Score
OCTR: Open carpal tunnel release
VIF: Variance inflation factor
